# Candidate DNA Barcode Tags Combined With High Resolution Melting (Bar-HRM) Curve Analysis for Authentication of *Senna alexandrina* Mill. With Validation in Crude Drugs

**DOI:** 10.3389/fpls.2018.00283

**Published:** 2018-03-13

**Authors:** Priyanka Mishra, Ashutosh K. Shukla, Velusamy Sundaresan

**Affiliations:** ^1^Plant Biology and Systematics, CSIR-Central Institute of Medicinal and Aromatic Plants, Research Centre, Bangalore, India; ^2^Biotechnology Division, CSIR-Central Institute of Medicinal and Aromatic Plants, Lucknow, India

**Keywords:** *Senna alexandrina*, herbal market, DNA barcode tags, real-time-PCR genotyping, high resolution melting curve analysis

## Abstract

*Senna alexandrina* (Fabaceae) is a globally recognized medicinal plant for its laxative properties as well as the only source of sennosides, and is highly exported bulk herb from India. Its major procurement is exclusively from limited cultivation, which leads to risks of deliberate or unintended adulteration. The market raw materials are in powdered or finished product form, which lead to difficulties in authentication. Here, DNA barcode tags based on chloroplast genes (*rbcL* and *matK*) and intergenic spacers (*psbA-trnH* and *ITS*) were developed for *S. alexandrina* along with the allied species. The ability and performance of the *ITS1* region to discriminate among the *Senna* species resulted in the present proposal of the *ITS1* tags as successful barcode. Further, these tags were coupled with high-resolution melting (HRM) curve analysis in a real-time PCR genotyping method to derive Bar-HRM (Barcoding-HRM) assays. Suitable HRM primer sets were designed through SNP detection and mutation scanning in genomic signatures of *Senna* species. The melting profiles of *S. alexandrina* and *S*. *italica* subsp. *micrantha* were almost identical and the remaining five species were clearly separated so that they can be differentiated by HRM method. The sensitivity of the method was utilized to authenticate market samples [Herbal Sample Assays (HSAs)]. HSA01 (*S. alexandrina* crude drug sample from Bangalore) and HSA06 (*S. alexandrina* crude drug sample from Tuticorin, Tamil Nadu, India) were found to be highly contaminated with *S*. *italica* subsp. *micrantha*. Species admixture samples mixed in varying percentage was identified sensitively with detection of contamination as low as 1%. The melting profiles of PCR amplicons are clearly distinct, which enables the authentic differentiation of species by the HRM method. This study reveals that DNA barcoding coupled with HRM is an efficient molecular tool to authenticate *Senna* herbal products in the market for quality control in the drug supply chain.

CIMAP Communication Number: CIMAP/PUB/2017/31

## Introduction

*Senna alexandrina* Mill. (syn. *Cassia Senna* L., *C. angustifolia* Vahl.) known under the trade name Tirunelveli *Senna* or Indian *Senna* is a globally recognized natural laxative drug recommended in Ayurveda, Siddha, Unani, Yoga, Naturopathy, and Homeopathy in India as well as in the pharmacopeias of United States, United Kingdom, Germany, and other counties (Irwin and Barneby, 1982; Bown, 1995; Al-Dakan et al., 1995; Singh, 2001). Tirunelveli *Senna* is named after a place in south India, which is a major hub for *Senna* cultivation in India. Apparently, the crop was introduced from North Africa and became naturalized in India. The plant is cultivated all over the subtropical tracts of India and is concentrated in the semi-arid parts of Tamil Nadu, Gujarat, and Rajasthan and exported under the brand name “Tirunelveli *Senna*" (Rama Reddy et al., 2015). The dried leaves and pods are the potent drug parts and contain anthraquinone glycosides known as *Senna* glycosides or sennosides (four types: A, B, C, and D). Sennosides A and B are the biologically active compounds of *Senna* that are used for their alleged purgative, expectorant, antidysentric, and carminative effects (Franz, 1993; Gupta and Pareek, 1995). The leaves and pods of the plant have been globally investigated for various therapeutic effects such as anti-mutagenic, anti-genotoxic, and anti-fungal properties (Lewis et al., 2005; Sultana et al., 2012; Cirillo and Capasso, 2015). India is presently the main source of cultivated *Senna* (recorded in over 10,000 ha) directed to the world market (Balasankar et al., 2013). An export volume of 15,975 metric tons, valued at USD 10 million, was achieved in 2012–2013, which has been growing steadily since then (*The Hindu*, Tuticorin Edn. dated 15.10.13).

The commercially available forms of the *Senna* drug include extracts and herbal supplements. *Senna* and its branded preparations, viz. GlaxennaR (Glaxo); Pursennid(R) (Sandoz); Helmacid with *Senna*(R) (Allenburrys) contain calcium sennosides, which are useful in treating habitual constipation. It is impossible to accurately identify constituent medicinal plant species in processed market products (such as dried raw drug, tablets, decoctions, and tea bags) through morphological characters. For the last decade, many DNA-based techniques, including DNA barcoding, have been adopted as versatile tools and have rapidly complemented the classical strategies being used for medicinal plant identification and authentication (Hebert et al., 2003; Li et al., 2011). DNA barcoding technology has attracted great attention and research interest for its practical applications in plant biodiversity assessment with its wider implementation in detection of adulteration in the herbal market (Mishra et al., 2016a). The diversity among DNA sequences used to identify taxa can be viewed as genetic barcode. Many studies have shown its potential in effectively identifying the constituent species in processed herbal medicines (Newmaster et al., 2013; Sarwat and Yamdagni, 2014; Mishra et al., 2017). However, the necessity of assessing a large number of sequences for developing a successful barcode tag corresponds to high cost of sequencing and sometimes restrains the application of DNA barcoding in developing countries (Osathanunkul et al., 2015).

Toward this end, high resolution melting (HRM) analysis of candidate DNA barcode marker resulting in the development of a DNA-based technological platform termed Bar-HRM has been adapted successfully. HRM curve analysis is a post real-time PCR based analytical technique, which measures the rate of dissociation of amplicons with increase in temperature. The method does not require the sequencing or hybridization analysis of the end products. The double stranded DNA is dissociated into single stranded DNA being monitored by fluorescence measurement of the intercalated dye included in the PCR reaction. The specific melting curve is obtained for each PCR product having significantly different melting temperature (*T*_m_) and peak locations (Reed and Wittwer, 2004). These raw curves are normalized via processing with HRM-based softwares, which define the changes in fluorescence on the basis of thermodynamic properties of the particular DNA product (Palais et al., 2005; Mishra et al., 2016a). Based on the amplification profile of HRM-designed primers, the melting kinetics facilitates the scanning of single nucleotide polymorphisms (SNPs), mutations, or methylation in the genomic signature of individual species (Wojdacz and Dobrovic, 2007; Toi and Dwyer, 2008; Wittwer, 2009). The BAR-HRM approach provides greater resolving power as compared to the conventional melting curve analysis through shape differentiation of the amplicons for the same *T*_m_ values. The study involves the designing of HRM specific primers based on the SNPs flanking regions in sequences derived from the plant barcoding markers. Thus for the development of Bar-HRM assays, a successful DNA barcoding study is necessary in the particular plant groups (Ganopoulos et al., 2013). Hitherto the published literature on Bar-HRM has demonstrated its high applicability in identifying adulterants in traded medicinal plants and its precision in identifying genuine drug species in the herbal market (Ganopoulos et al., 2012; Jiang et al., 2014; Kalivas et al., 2014; Buddhachat et al., 2015; Schmiderer et al., 2015; Song et al., 2016; Meistertzheim et al., 2017).

Tirunelveli *Senna* is a globally valued medicinal plant that has considerable commercial importance, but the brand name is jeopardized by-product substitution. *Alexandrian senna*, which has its basic origin in Sudan, is the same species as India *Senna* or Tirunelveli *Senna* (Schmelzer and Gurib-Fakim, 2008; Purushothaman et al., 2014; Mishra et al., 2016a). Due to high morphological disparity in the entire genus, the dried leaves of *S. alexandrina* are often mistaken for those of *S. auriculata* (Palthe *Senna*) and *S. obovata* (*S. italica* subsp. *micrantha*) commonly known as Dog *Senna*. The market survey revealed that *Senna* is nearly always adulterated with the leaves and pods of other *Senna* species. *S. alexandrina* is the only species in the genus, evaluated and reported for its laxative property with the presence of sennoside A and B. The use of any other *Senna* species in the herbal preparations without the active pharmacological principles reduces the efficacy of the *Senna* herbal formulations. In the present study, we attempted to develop the DNA barcode tags for *Senna* species with the ultimate goal to develop Bar-HRM markers for rapid authentication of *S. alexandrina* from its adulterants and to authenticate *Senna*-containing commercial products sold in the Indian market.

## Materials and Methods

### Plant Material

A total of 21 voucher samples derived from seven species of *Senna* were collected from different geographical locations of Tamil Nadu, Karnataka, and Uttar Pradesh (Supplementary Figure 1). These included three individuals of each of the following: *S. alexandrina*, *S. italica* subsp. *micrantha*, *S. spectabilis* subsp. *spectabilis*, *S. auriculata*, *S. uniflora, S. italica* subsp. *Italica*, and *S. tora* species. The authenticity of the samples was verified by Dr. V. Sundaresan, Senior Scientist, CSIR-Central Institute of Medicinal and Aromatic Plants (CSIR-CIMAP), Research Centre, Bangalore, using the taxonomical monographs, floras and through the herbarium vouchers from the Botanical Survey of India (BSI). The reference voucher samples were deposited in the herbarium maintained at CSIR-CIMAP, Lucknow and the obtained accession details are tabulated in Supplementary Table 1. Additionally, raw drugs samples were purchased from different drug stores and markets in Bangalore (Karnataka) and Tamil Nadu, and were randomly selected for testing. The market samples were coded as HSAs, vouchered accordingly and deposited in the herbarium of CSIR-CIMAP, Lucknow. The sample codes are HSA01, HSA02, HSA03, HSA04 from Bangalore and HSA05, HSA06, HSA07, HSA08, HSA09, and HSA10 from markets in Tamil Nadu (Supplementary Table 2).

### DNA Extraction, PCR Amplification, and Sequencing

Seven species with three individuals (*n* = 3) were used for study. Total genomic DNA from the field samples of reference species was isolated following the protocol described by Mishra et al. (2016b). The genomic DNA from commercial samples (HSA01–HSA10) was isolated with DNeasy^®^ PlantMini Kit (Qiagen, Valencia, CA, United States) using the protocols supplied with the kit. The quality of the DNA was checked by electrophoresis on a 0.8% agarose gel with standard markers and quantified by spectrophotometric analysis (NanoDrop, ND-1000, United States). The DNA was diluted to working concentration of 25–50 ng/μl for PCR amplifications. Five commonly used candidate DNA barcode markers were amplified from three individuals of each species with the established primers, which included; two coding cpDNA regions *rbcL* and *matK*; one non-coding cpDNA intergenic spacer region, *psbA-trnH* and the nrDNA regions, *ITS1* and *ITS2*. Details of primers and PCR conditions are listed in **Table 2**. PCR reactions were set up in a final volume of 50 μl with 1X Taq DNA polymerase buffer containing 1.5 mM Mgcl_2_, (Genei Bangalore, India), 200 μM of each dNTP (Genei Bangalore, India), 5–10 pmol each of forward and reverse primers, 1 Unit of Taq DNA polymerase (Genei Bangalore, India) and 25–50 ng of template DNA. Successful amplicons were analyzed through electrophoresis on a 2% agarose gel. All reactions were performed in triplicates. Subsequently products of target molecular weight were purified with a Nucleospin PCR purification kit (Macherey-Nagel–07/2014, Rev.03) according to the supplied protocol and rechecked through electrophoresis on a 2% agarose gel. The PCR amplicons were sequenced from both ends through Sanger sequencing, using the Big Dye Terminator v3.1 Cycle Sequencing Kit (Applied Biosystems, Inc., Foster City, CA, United States) on an ABI 3130 XL genetic analyzer (Applied Biosystems, Inc., Foster City, CA, United States).

### Databasing and Sequence Analysis

Specimen data for each barcode region were deposited in the Barcode of Life Data Systems (BOLD)^1^ (Ratnasingham and Hebert, 2007) under the project CRCBS-Barcode marker for *Senna* authentication (Supplementary Table 1). All the related data are publicly accessible under the dataset DS-CIMAP^2^. Lab-generated barcode sequences were deposited to the GenBank (Benson et al., 2013) under accession numbers listed in Supplementary Table 1. The electropherograms obtained for each region were base-called using PHRED vII (Ewing and Green, 1998). The proofreading of sequencing peaks and contig assembly were done in Sequencher v5.4.6 (Gene Codes Corporation, Ann Arbor, MI, United States). Finally, the contigs were analyzed through NCBI BLASTN 2.2.1+ (Zhang et al., 2000; Morgulis et al., 2008) and loaded on to BOLD using Identification Request for accessing their identity percentage with other similar sequences in database. All the barcode sequences were greater than 300 bases in length and free from contamination. The sequences were then aligned with Muscle 3.8.31 on the EMBL-EBI website^3^ under default parameters and alignments were adjusted manually in BioEdit v7.1.3.0 (Hall, 1999). The sequences were trimmed at both the ends to remove the primer sequences and the variable sites were reconfirmed using the original trace files.

### *In Silico* DNA Barcode Analysis and HRM Primer Design

The five candidate DNA barcode markers and their 20 possible combinations in multigene and tiered barcoding approaches were evaluated based on the methods proposed by the consortium for the barcode of life (CBOL). Candidate diagnostic nucleotides to classify sequences from specimens to species using a set of classification rules were identified using character-based machine learning approach in the program BLOG2.0 (Bertolazzi et al., 2009; Weitschek et al., 2013). The different barcode datasets used in this study were subjected to 90% slicing within species-level. The maximum iterations was set to 500 (GRASPITER = 500) with maximum given time of 5 min for complete analysis (GRASPSECS = 300). Among the derived logic formula sets, the one with the lowest false positive rate against the reference dataset was used as identification basis in *Senna*.

Bayesian-inference (BI) analysis was performed on *ITS* marker using MrBayes v.3.2.2 (Ronquist et al., 2012) at the CIPRES Science Gateway^4^. The best fit substitution model GTR+G was selected for analysis using the jModelTest v2.1.7 (Posada, 2008). The Bayesian analysis with the metropolis-coupled Markov Chain Monte Carlo (MCMC) was run for 10,000,000 generations, saving at every 1000th generation (the first 25% of trees were discarded as burn-in). The generations were checked until the average deviation of split frequencies reached under 0.01 and the potential scale reduction factor (PSRF) for all parameters approached 1.0. Convergence of runs was assessed using Tracer v. 1.6 (Rambaut et al., 2014) to generate a consensus tree with Bayesian posterior probabilities (PP) values. The values ≥0.95 were considered and included for each marker and concatenated topologies. To verify the results of the HRM analysis, the neighbour joining (NJ) tree-based method was used for species identification analyses. The NJ tree was constructed using MEGA6.0 with Kimura-2 parameter (K2P) model. The reliability of each node was assessed by performing a bootstrap analysis set to 1000 pseudo-replicates (Felsenstein, 1988).

Based on a previous study (Mishra et al., 2016b) and the results from the present dataset, *ITS1* barcode reflected significant sequence divergence among the seven *Senna* species and were thus selected for subsequent HRM analysis. Conserved regions flanking the variable sites were identified visually and HRM-suited potential primers were designed with AlleleID (v.7.7, Premier Biosoft International, Palo Alto, CA, United States) using its SYBR Green Design function. The target amplification was set between 100 and 250 bases and primer length was set to 15 and 30 bases with an estimated melting temperature of 55.0 ± 5.0°C. The internal primers were screened for optimal coverage of polymorphic sites and conserved sequence at primer sites. The designed primers were analyzed using the IDT OligoAnalyzerv3.1 tools^5^ for the primer properties, hairpins and self/cross hybridization. The primers were verified using the Primer-BLAST NCBI to ensure specificity and were synthesized through Eurofins Analytical Services India Pvt. Ltd. (Bangalore, India).

### Real-Time PCR Amplification and HRM Analysis

Real-time PCR amplification followed by DNA melting and fluorescence measurements was performed on the StepOnePlus^TM^ Real-Time PCR System (Applied Biosystems, Inc., Foster City, CA, United States). The real-time PCR assays consisted of 5 μl of 2x MeltDoctor HRM Master Mix (Applied Biosystems, Inc., Foster City, CA, United States), 0.2 μl of 10 mM forward and reverse primers, 1 μl (1–50 ng) of genomic DNA and DNAse-free water to make up the final volume to 10 μl. Positive (containing a known amount of genomic DNA from each species) and negative controls were included. PCR runs were conducted using an initial denaturing step at 95°C for 5 min followed by 35 cycles of 95°C for 30 s, 57–60°C for 30 s and 72°C for 20 s, then a final extension step of 72°C for 2 min with collection of fluorescence signal at the end of each cycle. For HRM analysis, the PCR products were denatured at 94°C for 10 s and then annealed at 50°C for 15 s to randomly form DNA duplexes. The melting analysis was performed with the temperature increasing from 60 to 95°C at a ramp rate of 0.1°C/s. Fluorescence data were acquired at the end of each melting phase and processed using High Resolution Melt Software v3.0 (Applied Biosystems, Inc., Foster City, CA, United States). All reactions were done in triplicates and reference samples for HRM profiles analysis were included. The samples providing cycle threshold (*C*_t_) values below 30 were considered suitable for HRM analysis. The -dF/dT (negative derivative of fluorescence F over temperature T) curve were plotted to derive the characteristic *T*_m_ for every species; the normalized raw curve depicted the decrease in fluorescence with increasing temperature. To normalize the raw melting curves, pre- and post-melt normalization regions were set and adjusted to define the temperature boundaries of the used plots. The characteristic melting temperatures (*T*_m_) were recorded for each species of the *Senna* clade. *S. alexandrina* was set as a reference species. The developed Bar-HRM method was authenticated on the commercial *Senna* samples for detection of constituent species. DNA extracted from the raw drug powder of *S. alexandrina* was pooled with DNA of other six *Senna* species, viz. *S. italica* subsp. *micrantha*, *S. spectabilis*, *S. auriculata*, *S. uniflora, S. italica* subsp. *Italica*, and *S. tora* in percentage of 4, 8, 12, 25, and 50% w/w and tested for their specificity.

## Results

### PCR Amplification and Sequencing Success Rate

Seven potential species of the genus *Senna* representing a total of 21 individuals were successfully amplified and sequenced using five DNA barcodes, viz. *rbcL*, *matK*, *psbA-trnH*, *ITS1*, and *ITS2* with 100% PCR and sequencing success rate (**Table 1**). The present study generated 105 new sequences, which were submitted to BOLD database and GenBank (Supplementary Table 1) and their diagnostic characteristics have been tabulated in **Table 1**. The PCR amplicons of all the five barcodes ranged according to the average size of the respective marker (**Table 2**). NCBI BLAST hits of all the seven species shared maximum similarity ∼98–100% with other species and to other genera of Cassiinae group. *ITS* sequences ranged from 607 to 721 bases with 738 aligned sites comprising of 304 variable and 303 parsimony informative sites. The sequences of complete *ITS* regions were annotated and trimmed to the regions of *ITS1, 5.8S* and *ITS2*. The sequence length of *ITS*2 ranged from 457 to 462 bases with 474 aligned sites. The numbers of variable and parsimony informative sites were 154. Both the markers showed several indels in the range of 1–4 bases within the aligned region. The coding regions *rbcL* and *matK* were highly conserved with 682/705 sites 726/785 sites, respectively, and were without indels. The intergenic spacer *psbA-trnH* showed high sequence length variation with 341–384 bases and the number of aligned sites were 437. The aligned region comprised several indels of 1–4 bases and 359 conserved sites.

**Table 1:** Sequence characteristics of the five DNA barcode markers evaluated in this study.

Parameters assessed	DNA barcode marker				
	*rbcL*	*matK*	*psbA-trnH*	*ITS*	*ITS2*
Number of individuals	21	21	21	21	21
PCR success (%)	100	100	100	100	100
Sequencing success (%)	100	100	100	100	100
Sequence length (bases)	705	785	341–384	607–721	457–462
Aligned length (bases)	705	785	437	738	474
No. of variable sites	23	59	78	304	154
No. of indels	0	0	15	27	21
Proportion of parsimony informative sites	21/705	53/785	71/437	303/738	154/474
Pairwise identity (%)	99.1	97.7	84.4	76.5	85.7

**Table 2:** Primers used for amplification and sequencing for DNA barcoding and HRM amplifications of reference species.

Region	Primer name	Sequence (5′–3′)	Thermal cycling conditions	Mean size	Reference
*rbcL*	1F 724R	5′-ATGTCACCACAAACAGAAAC-3′ 5′-TCGCATGTACCTGCAGTAGC-3′	95°C 2 min; (35 cycles: 94°C 1 min; 55°C 30 s; 72°C 1 min); 72°C 7 min	715 bp	Kress et al., 2005
*matK*	390F 1326R	5′-CGATCTATTCATTCAATATTTC-3′ 5′-TCTAGCACACGAAAGTCGAAGT-3′	95°C 2 min; (30 cycles: 94°C 1 min; 48°C 30 s; 72°C 1 min); 72°C 7 min	856 bp	Cuénoud et al., 2002
*psbA-trnH*	psbAF trnHR	5′-GTTATGCATGAACGTAATGCTC-3′ 5′-CGCGCATGGTGGATTCACAATCC-3′	94°C 5 min; (35 cycles: 94°C 1 min; 55°C 30 s; 72°C 1.5 min); 72°C 7 min	400 bp	Zheng et al., 2014
*ITS*	ITS5a ITS4	5′-CCTTATCATTTAGAGGAAGGAG-3′ 5′-ATGCGATACTTGGTGTGAAT-3′	94°C 5 min; (30 cycles: 94°C 1 min; 50°C 1 min; 72°C 1.5 min); 72°C 7 min	704 bp	Kress et al., 2005
*ITS*2	S2F S3R	5′-TCCTCCGCTTATTGATATGC-3′ 5′-GACGCTTCTCCAGACTACAAT-3′	94°C 5 min; (40 cycles: 94°C 30 s; 56°C 30 s; 72°C 45 s); 72°C 10 min	450 bp	Chen et al., 2010
HRM	SIM026HRMF	5′-CGAAGCCATTAGGTTGAG-3′	94°C 5 min; (35 cycles: 95°C 30 s; 57–60°C 30 s; 72°C 20 s); 72°C 2 min	79 bp	Through this study


HRM	SIM026HRMR	5′-ATTGAACGGAGGGATGAC-3′			
HRM	SU029HRMF	5′-CAAGGAACCCAAACGAAC-3′		78 bp	
HRM	SU029HRMR	5′-ACATCATTTCCGTGGAAGA-3′			
HRM	ST030HRMF	5′-TTATCAATTAGAGGAAGGAG-3′		112 bp	
HRM	ST030HRMR	5′-GAGTGTTTCAACCAATTC-3′			
HRM	SII031HRMF	5′-CAAGGAACAGATAAATGGA-3′		97 bp	
HRM	SII031HRMR	5′-GAGAGTCATTGTGGATAC-3′			

### Efficiency of Barcoding Regions for Species Identification in *Senna*

**FIGURE 1:**
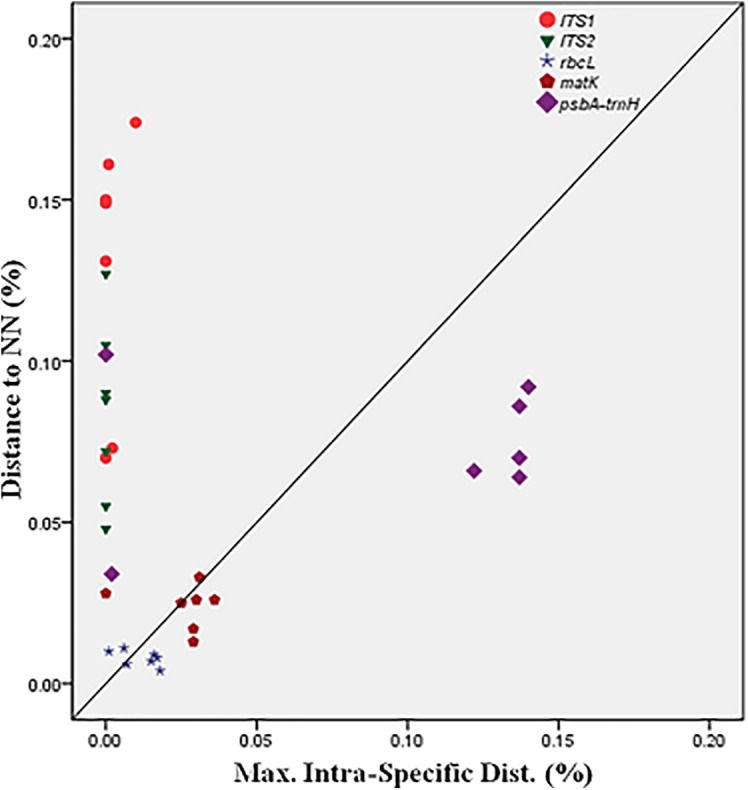
Barcode gap plot for the five individual barcodes.

BOLD-based genetic distance analysis of the four barcode regions *matK, ITS1, ITS2* and *psbA-trnH* revealed that mean intra-specific distances were less than the distance to nearest neighbour (NN) of each *Senna* species. Individuals of *S. italica* subsp. *italica* exhibited maximum intra-specific divergence of 0.77–16.03%. The highest divergence range recorded is also due to inclusion of the individuals of subsp. *micrantha. S. auriculata* and *S. tora* share a maximum identity of 0.14% in terms of nearest neighbor (**Table 3**). Among the single barcode markers, only the *ITS* region resulted in the existence of a clear barcode gap, which is ideal for species identification (**Figure 1**). Besides, the two-barcode combination increased the resolution percentage in most of the tested combinations. *S. alexandrina* and *S. italica* subsp. *micrantha+italica* recorded the lowest NN distance of 5.74% among them, which reflects the high genomic similarity among them. The nuclear region *ITS1* and *ITS2* showed maximum inter-specific distances (4.97–19.34%) among all the seven species, thereby qualifying as potent marker for discriminating the species of genus *Senna*. However, the coding region *rbcL* showed only 0.14–0.43% divergence with any of the nearest neighbor among all seven species (**Table 3**). **Figure 1** depicts the scatter plot of the maximum intra-specific distances against the NN distances to confirm the existence and magnitude of the barcode gap for all five candidate barcodes. Maximum intra-specific distances were less than 2% in all the species, except *S. spectabilis* (2.73%) and *S. italica* subsp. *italica* (16.03%). Based on the utility of individual markers, *ITS1* and *psbA-trnH* were the favorable choices in the genus *Senna* and the regions were combined with other markers to assess their resolution rate (**Table 4**).

**Table 3:** Mean and maximum intra-specific and nearest neighbor (NN) distance for all the species using candidate barcodes.

Barcode	Species	Mean intra-specific distance	Max intra-specific distance	Nearest neighbor	Distance to NN
*rbcL*	*Senna alexandrina*	0	0	*Senna tora*	0.43
	*Senna auriculata*	0	0	*Senna tora*	0.14
	*Senna italica*	1.04	1.72	*Senna alexandrina*	0.28
	*Senna spectabilis*	0.48	0.71	*Senna tora*	0.43
	*Senna tora*	0	0	*Senna auriculata*	0.14
	*Senna uniflora*	0.38	0.57	*Senna auriculata*	0.28
*matK*	*Senna alexandrina*	0	0	*Senna auriculata*	1.68
	*Senna auriculata*	0	0	*Senna uniflora*	1.29
	*Senna italica*	0.42	0.77	*Senna spectabilis*	1.29
	*Senna spectabilis*	1.07	2.73	*Senna italica*	1.29
	*Senna tora*	0	0	*Senna auriculata*	2.46
	*Senna uniflora*	0.09	0.13	*Senna auriculata*	1.29
*psbA-trnH*	*Senna alexandrina*	1.94	2.5	*Senna italica*	5.71
	*Senna auriculata*	0	0	*Senna uniflora*	0.84
	*Senna italica*	4.34	10.23	*Senna alexandrina*	5.71
	*Senna spectabilis*	0	0	*Senna auriculata*	3.42
	*Senna tora*	0.2	0.29	*Senna auriculata*	3.34
	*Senna uniflora*	0	0	*Senna auriculata*	0.84
*ITS1*	*Senna alexandrina*	0	0	*Senna italica*	7.27
	*Senna auriculata*	0	0	*Senna uniflora*	7.73
	*Senna italica*	2.13	15.75	*Senna alexandrina*	7.27
	*Senna spectabilis*	0	0	*Senna italica*	19.34
	*Senna tora*	1	1.5	*Senna auriculata*	17.88
	*Senna uniflora*	0	0	*Senna auriculata*	7.73
*ITS2*	*Senna alexandrina*	0	0	*Senna italica*	5.74
	*Senna auriculata*	0	0	*Senna uniflora*	4.97
	*Senna italica*	5.59	16.03	*Senna alexandrina*	5.74
	*Senna spectabilis*	0	0	*Senna italica*	14.81
	*Senna tora*	0	0	*Senna auriculata*	11.86
	*Senna uniflora*	0	0	*Senna auriculata*	4.97

**Table 4:** Species identification rates in % (correctly identified/misidentified/not identified) using two different classification methods for each of the five barcodes and their combinations.

	Taxon DNA		
Barcode marker/markers	Best match (%)	Best close match (%)	BLOG
*rbcL*	100/0/0	95.23/0/4.76	100/0/0
*matK*	90.47/9.52/0	90.47/9.52/0	85.71/0/14.29
aaagray!60*psbA-trnH*	aaagray!60100/0/0	aaagray!60100/0/0	aaagray!60100/0/0
aaagray!60*ITS1*	aaagray!60100/0/0	aaagray!60100/0/0	aaagray!60100/0/0
*ITS2*	100/0/0	95.23/0/4.76	100/0/0
*rbcL+matK*	95.23/0/4.76	95.23/0/4.76	85.71/0/14.29
*rbcL+psbA-trnH*	100/0/0	100/0/0	85.71/0/14.29
*rbcL+ITS1*	100/0/0	95.23/0/4.76	100/0/0
*rbcL+ITS2*	100/0/0	95.23/0/4.76	100/0/0
*matK+psbA-trnH*	100/0/0	100/0/0	100/0/0
*matK+ITS1*	100/0/0	100/0/0	100/0/0
*matK+ITS2*	100/0/0	100/0/0	85.71/0/14.29
aaagray!60*psbA-trnH+ITS1*	aaagray!60100/0/0	aaagray!60100/0/0	aaagray!60100/0/0
aaagray!60*psbA-trnH+ITS2*	aaagray!60100/0/0	aaagray!60100/0/0	aaagray!60100/0/0
*ITS1+ITS2*	100/0/0	95.23/0/4.76	100/0/0
*rbcL+matK+psbA-trnH*	100/0/0	100/0/0	85.71/0/14.29
*rbcL+matK+ITS1*	100/0/0	100/0/0	100/0/0
*rbcL+matK+ITS2*	100/0/0	100/0/0	85.71/0/14.29
*rbcL+psbA-trnH+ITS1*	100/0/0	100/0/0	100/0/0
*rbcL+psbA-trnH+ITS2*	100/0/0	100/0/0	100/0/0
*rbcL+ITS1+ITS2*	100/0/0	95.23/0/4.76	100/0/0
*matK+psbA-trnH+ITS1*	100/0/0	100/0/0	100/0/0
*matK+psbA-trnH+ITS2*	100/0/0	100/0/0	85.71/0/14.29
*matK+ITS1+ITS2*	100/0/0	100/0/0	100/0/0
*psbA-trnH+ITS1+ITS2*	100/0/0	100/0/0	100/0/0

*TaxonDNA: Best match and best close match results. Not identified rates are summed over the “Ambiguous” and “No match” categories. BLOG: percentage correct classification for test file, using 90% slicing at species level (refer to materials and methods for detailed analysis). The highest success rate for the preferred barcoding options in *Senna* are highlighted in gray*.

All five candidate barcodes and their 20 possible combinations were compared with TaxonDNA and BLOG to conclude on their discrimination percentage. All the barcoding datasets represented equal number of individuals corresponding to respective species and the rates of correctly identified, misidentified and not identified percentage were recorded for each datasets. Averaged over both the methods, *ITS1* and *psbA-trnH* produced the highest success rate (100%) among the tested single barcodes (**Table 4**). The coding regions *rbcL* and *matK* resulted in very poor discrimination success for single as well the two-combination barcodes. However, supplementing the combination of *rbcL+matK* with the non-coding *ITS1*region, itself being at the first position, afforded 100% correct identification (100/0/0) through both TaxonDNA and BLOG based methods. Thus the tiered approach of barcoding proved a promising way to barcode the species of the genus *Senna*.

### Evolutionary Relationships in *Senna*

**FIGURE 2:**
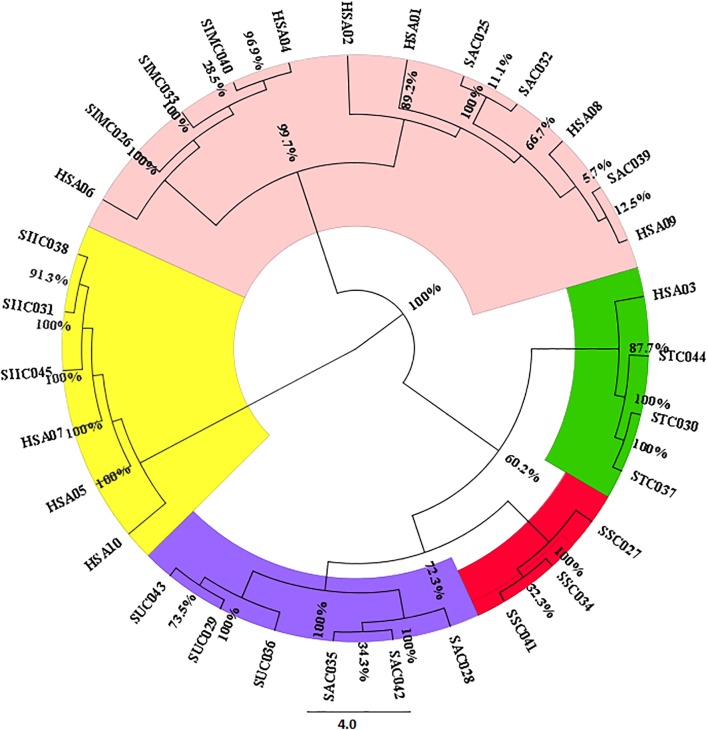
Neighbor-joining based clustering pattern of seven *Senna* species and their commercial samples inferred from the *ITS1* marker; Species codes: SAX – *S. alexandrina*, SIM – *S. italica* subsp. *micrantha*, SSP – *S. spectabilis*, SAU – *S. auriculata*, SU – *S. uniflora*, ST – *S. tora*, SSI – *S. italica* subsp. *italica*, HSA01-10: market samples.

For estimating the evolutionary divergences among the species of the genus *Senna*, we employed character-based methods on all the barcode regions carried out using the BI model in MrBayes. The consideration of barcode marker based on the computational phylogenetics depicted similar hypothesis in agreement with the method based on the presence of barcoding gap. As shown in the phylogenetic tree the most favorable barcode dataset *ITS1* presented highest level of discrimination at the species level. Bootstrap values clustered at the species level ranged from 64 to 100%. In particular the individuals belonging to same species maintained the species monophyly reflecting no intra-specific divergence among them. *S. alexandrina* and *S. italica* subsp. *micrantha* framed to be 100% similar (Supplementary Figure 2), which is consistent with the results from the BLAST analysis. Also the market samples of raw drug of *S. alexandrina* showed the presence of *S. italica* subsp. *micrantha*, when analyzed at the molecular level (**Figure 2**). Based on the indel polymorphism, we obtained marker nucleotides that can be used for species discrimination. Species-specific SNP positions and indel mutations detected in the *ITS* regions (total 143 sites) allowed the simultaneous discrimination of *S. italica* subsp. *italica* and *S. alexandrina* with transition mutations C ↔T and G ↔ A at 448 bp and 584 bp positions in the aligned length of antisense primer region. At the large polytomy, all the sister species of *S. alexandrina* shared maximum identity with 77–100% posterior probabilities. Individuals of *S. italica* subsp. *italica* framed the nodal cluster at the base of the tree (Supplementary Figure 2).

### Reproducibility and Specificity of the HRM Method

**FIGURE 3:**
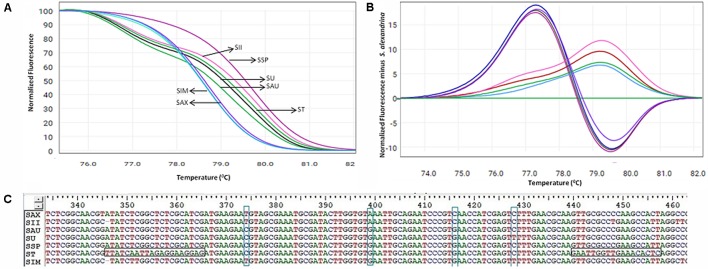
High resolution melting analysis using the HRM primers for wild samples **(A)** Normalized curves of the seven *Senna* species; Species codes: SAX – *S. alexandrina*, SIM – *S. italica* subsp. *micrantha*, SSP – *S. spectabilis*, SAU – *S. auriculata*, SU – *S. uniflora*, ST – *S. tora*, SSI – *S. italica* subsp. *italica*. **(B)** Difference plot curves of the amplicons using *S. alexandrina* as reference genotype. **(C)** Multiple sequence alignment of the amplified region of primer ST030HRMF/R. Primer regions are highlighted with horizontal rectangular boxes and nucleotide differences are highlighted with vertical rectangular boxes.

Based on the ability and performance of *ITS1* DNA barcodes, the developed *ITS*-HRM primers (**Table 2** and Supplementary Figure 3) that allowed the successful discrimination of the *S. alexandrina* from its allied species *S. italica* subsp. *micrantha*, *S. spectabilis*, *S. auriculata*, *S. uniflora, S. italica* subsp. *Italica*, and *S. tora* (**Figure 3A** and **Table 5**). Negative controls did not indicate any presence of plant species template. All the replicate specimens per species resulted in nearly similar *C*_t_ values and shapes of melting curves, which were further confirmed by the sequencing of the end products. *S. alexandrina* and *S. italica* subsp. *micrantha* depicted similar *C*_t_ values resulting in similar amplification efficiencies among species. Further the identifications derived by the HRM assays were confirmed by sequencing of the region of all the analyzed DNA extracts. The corresponding sequences were submitted to GenBank under the accession numbers listed in Supplementary Table 1, and the interpretations of both the sequencing and HRM profiles are found in **Table 5**. Both the techniques concluded 80–100% match concerning the targeted species. For samples SIM026 and SII031, which are difficult to discriminate visually by morphological classifications, the developed melting profile assigned them as the correct plant species consistent with the sequencing results (**Figure 3**).

**Table 5:** The values of melting temperature (°C) gaining for High Resolution Melting (HRM) of *Senna* species and sequence identity through BLAST analysis.

Species	Melting temperature	*T*_m_ (°C)	BLAST identity
*Senna alexandrina*	78.80 ± 0.05	*Senna alexandrina*
*Senna italica* subsp. *micrantha*	78.77 ± 0.07	*Senna alexandrina*
*Senna spectabilis* subsp. *spectabilis*	79.62 ± 0.15	*Senna spectabilis* subsp. *spectabilis*
*Senna auriculata*	78.91 ± 0.08	*Senna auriculata*
*Senna uniflora*	79.02 ± 0.06	*Senna uniflora*
*Senna tora*	79.31 ± 0.05	*Senna tora*
*Senna italica* subsp. *italica*	79.45 ± 0.10	*Senna alexandrina*

### Sensitivity and Discrimination Power of HRM Primer Assays

Aligned sequences of seven *ITS* markers were used to screen the suitable primer sets for HRM analysis. As a result we were able to find four primer sets specific to species *S. italica* subsp. *micrantha* (SIM026HRMF/R), *S. uniflora* (SU029HRMF/R), *S. tora* (ST030HRMF/R) and *S. italica* subsp. *italica* (SII031HRMF/R), which could amplify 79 bp, 78 bp, 112 bp (Supplementary Figure 3), and 97 bp amplicons, respectively. The three best primer sets SIM026HRMF/R, ST030HRMF/R, and SII031HRMF/R were able to amplify with all the targeted species, while the primer set SU029HRMF/R was found to work for *S. uniflora, S. auriculata*, and *S. tora*, only. Thus the previous three primer sets were found to be suitable for the HRM analysis in *Senna* due to their universality among the species. The amplified PCR product of these primer sets were found plausible with further evaluation, which contains the variations of nucleotide sequences when compared within the target species.

Our HRM analysis using the primer pair ST030HRMF/R distinguished between the species of *Senna* clade with characteristic *T*_m_ values recorded for each species (**Figures 3A,B**). *S. italica* subsp. *italica* differed in the PCR product size from the other six species due to the presence of indels (at positions 345 and 346) among them, resulting in a *T*_m_ value (79.45°C) different from that of *S. alexandrina* (*T*_m_ 78.80°C). Also the change of nucleotides from T to C between them (**Figure 3C**) resulting in their differences in *T*_m_. Amplicon from *S. spectabilis* and *S. uniflora* showed the lowest *T*_m_ value difference (79.62°C and 79.02°C, respectively) due to almost identically sized PCR products. The change of nucleotides C to A at position 355 in the forward primer region in the species *S. alexandrina* and *S. italica* subsp. *micrantha* from the rest of the species, resulted in almost identical *T*_m_ values (78.80°C and 78.77°C, respectively) for both the species. To better visualize the small difference between the individual melting curves, HRM software was used to calculate a difference plot for each species (**Figure 3B**). *S. alexandrina* was used as a reference species for genotyping with its melting curve as the baseline. The difference was obtained by subtracting the difference graph area of the reference species from the rest of the species derived melting curve. The genotype confidence level was measured on the cut-off value of 90% to assign a specific genotype for each barcode region. Thus the *ITS1* barcode coupled to HRM primer pair amplified all the seven species with sufficient discrimination and confidence level (**Figure 3B**).

### Evaluation and Quantification of Commercial Crude Drug Samples of *Senna*

**FIGURE 4:**
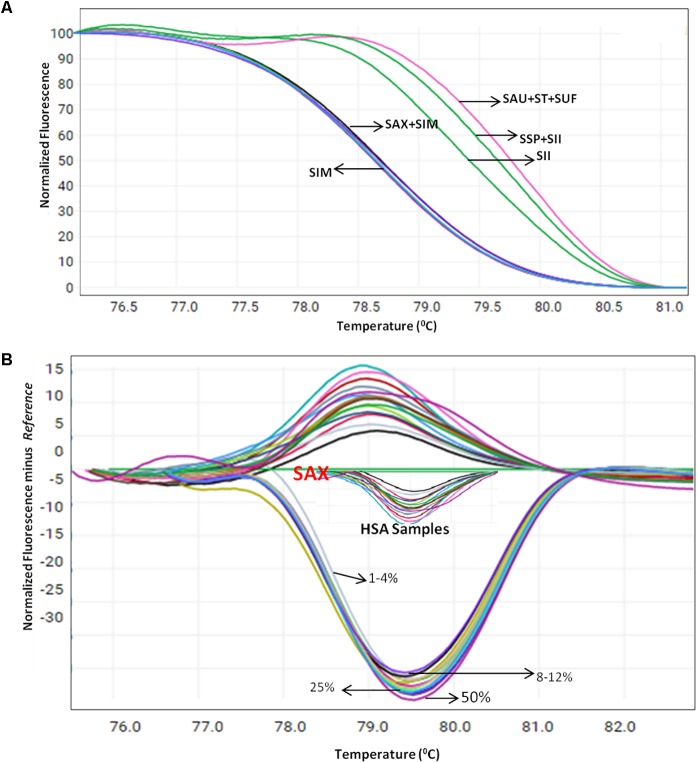
High resolution melting analysis using the HRM primers for quantification of admixture in wild and commercial samples pooled in various percentages **(A)** Normalized curves of the seven *Senna* species **(B)** Difference plot curves of the amplicons using *S. alexandrina* as reference genotype. Species codes: SAX – *S. alexandrina*, SIM – *S. italica* subsp. *micrantha*, SSP – *S. spectabilis*, SAU – *S. auriculata*, SU – *S. uniflora*, ST – *S. tora*, SSI – *S. italica* subsp. *italica*.

Herbal plant identification becomes more challenging when the plants are in processed or dry form, which often is due to the high morphological similarity of the drug part with respect to its adulterants (Supplementary Figure 4). To test the blend of other species in *S. alexandrina* samples, HRM coupled with the *ITS1* barcode method was employed on the pooled DNA of *S. alexandrina* contaminated with the other six *Senna* species. The contamination percentage was measured as 1–50%. Consequently, when the sample of *S. alexandrina* was mixed with the *S. italica* subsp. *micrantha* species in range of 1–10%, the *T*_m_ values of PCR product started deviating gradually toward *S. italica* subsp. *micrantha* species (**Figure 4A**). The identification of the contaminant was not easy when mixed in range of 1–5%. However, the differentiation slightly increased above 6%, to detect the admixture of other species in the market samples of *S. alexandrina*. The limit of detection was recorded between 8 and 12%. Besides, 10 commercial samples (HSA01–HSA10) from local markets were also tested with the method to confirm their specificity. *T*_m_ values of PCR product from samples HSA01 and HSA06 were found to be in same range (**Figure 4B**). In contrast, both the samples presented similar melting curve profiles and cannot be differentiated further. Both the *Senna* samples were found to be contaminated with plant species *S. italica* subsp. *micrantha*. Sample HSA08 and HSA09 had *T*_m_ values almost identical to the *T*_m_ value recorded in the field samples of *S. alexandrina*. Thus both the samples were putatively found to be *S. alexandrina*. Sample HSA08 was found to be contaminated with *S. tora*, with *T*_m_ values of 79.30°C. Thus the present investigation resulted in the development of a robust and rapid method to detect the admixture of *S. alexandrina* raw drugs contaminated with other *Senna* species, based on their unique melting curves (**Figure 4A**).

### Validation of HRM Results With DNA Sequencing

The findings from the HRM results were further validated with bidirectional sequencing of the HRM-PCR products. All the obtained sequences were checked for their similarity with the BOLD database. The BLAST analysis of the HSA sample sequences were found to be in congruence with the results of the HRM analysis. A phylogenetic tree was constructed on the sequence dataset employing the NJ cluster algorithm (**Figure 2**). HSA04 and HSA06 were found to be highly contaminated with *S. italica* subsp. *micrantha*. HSA03 was mixed up with *S. tora* and *S. uniflora* with 60% contamination. HSA01, 02, 08, and 09 were the putative *S. alexandrina* species, while HSA05, 07, and 10 were found to be clustered with *S. italica* subsp. *micrantha*. The corresponding sequences have been submitted to DNA barcoding database BOLD for species authentication. These results demonstrate the usefulness of *ITS1* barcode in species authentication in the genus *Senna*.

## Discussion

Current developments in the identification and authentication of plant taxa in herbal market are moving toward using a combination of approaches. DNA barcoding has been established as a straightforward solution to complex problem in providing a way to confirm the authentication of raw plant material and establish a level of quality assurance within the market (Mishra et al., 2016a, 2017). Recently, the technology has gained ample attention with the complement of real-time PCR-based analysis of melting curve termed as HRM for detection of contaminants in herbal samples. It has become a reliable and highly useful molecular technique in many fields, In comparison to high-throughput sequencing tools for any sequence analysis, HRM method requires a minimum of processing steps and cost, but its sensitivity varies with the number of samples (with/without mutations), where the delineation of the curves becomes more difficult and positive results require DNA sequencing for validation (Reed et al., 2007; Cousins et al., 2012). Thus the prior characterization of the target plant group through DNA barcoding studies is a prerequisite. Hitherto, several studies have reported its potential application in different plant species (Ganopoulos et al., 2012; Kalivas et al., 2014; Schmiderer et al., 2015; Song et al., 2016; Sun et al., 2016, 2017; Meistertzheim et al., 2017; Mishra et al., 2017). *S. alexandrina* is well known for its laxative properties and high export value as a bulk herb in trade from India. The plant is widely sourced from the wild populations and in the absence of sufficient raw drug material the species is often substituted or adulterated with other species of genus *Senna*. However, till date, taxonomical keys are the only tool available to characterize these plant species in the herbal market. We made an effort toward the development of a species-specific SCAR marker-based tool to authenticate the genuine *Senna* species to detect adulteration in the market (Mishra et al., under communication).

The relative usefulness of each of the five tested loci amplified from seven different *Senna* species was analyzed by comparing their amplification and sequencing success rates among the tested species. PCR amplifications and sequencing success rates were 100% for *rbcL*, *matK*, *psbA-trnH*, *ITS*, and *ITS2*. From among the tested plastid and nuclear loci, *ITS1* had the highest efficiency as a single locus in identification of species in *Senna* (**Figure 1**). *ITS1* showed significantly lower GC content having positive effect on PCR and sequencing efficiencies. The two barcodes *rbcL* and *matK* had the lowest discriminatory power as a single locus, which limit their utility in *Senna* despite being the powerful regions of barcoding in other plant groups. Both the regions failed to discriminate between the species and the resulting phylogenetic tree showed huge over-mixing of individuals with poor clade support. The greatest distance to NN was 0.43% only with *S. italica* and *S. tora*. Comparably *matK* gene showed much better resolution of 2.46% of with *S. auriculata* (**Table 3**). The suitability of chloroplast region *rbcL* at the specific levels of molecular evolution had mostly been controversial owing to its ∼1430 bp length. For clear species discrimination, the entire region needs to be sequenced, which limits its use as a barcoding sequence. Besides, the coding plastid gene *matK* currently does not have one primer that works for all plant species. Both the regions failed to retrieve the envisaged regions with the specified universal primer pairs, failing to fulfill the primary requirement of DNA barcoding. The primer pairs of *ITS1* investigated in studying the Cassinae group generally resulted in the successful amplification of amplicons from remarkably more species than those of *ITS2*. Following the results of large-scale meta-analysis *ITS1* claimed to be better DNA barcode than *ITS2* (Mishra et al., 2016b). Moreover, due to lower rate of nucleotide substitution in the chloroplast genome as compared to the nuclear genome, it provides less variable sites for analysis. Besides the evolution of aneuploidy and introgression in plant speciation hurdles the use of only the chloroplast genome for barcoding (Wolfe et al., 1987).

To evaluate the applicability of the HRM method in the characterization of *Senna* species, the *ITS1* barcode was selected as the target region. The HRM method applied with *ITS1* barcode resulted in similar melting curves for the *S. alexandrina* genome, irrespectively of whether the DNA was sourced from the natural population, crude drug material or finished herbal products. Interspecific heterogeneity in the *ITS1* region allowed sufficient discrimination of all seven *Senna* species examined (**Figures 2**, **3**). All tested species produced a single amplicon also verified by gel electrophoresis of the end products. However, raw melting profiles of few samples consisted of two peaks, of which the lower one was at low temperature and vice versa. One possible reason is that the melting behavior of the amplicons depends on the sequence length and GC content (Reed et al., 2007). Melting in the AT-rich region results in a peak at lower temperature whereas melting in GC-rich regions results in peak at higher temperature. Therefore the amplicon sequences were checked using an online calculator^6^ for the GC rich segment. The plotting pattern showed 36.3–56.8% GC segment lies in within the starting region till ∼80 bases. Also the two very closely allied species *S. alexandrina* and *S. italica* subsp. *micrantha* resulted in very similar *T*_m_ values due to low interspecific divergence but can still be distinguished by their difference plot (**Figure 3A** and **Table 5**). Species admixture in commercial samples of *Senna* has also been reported in the earlier study (Seethapathy et al., 2014) based on DNA barcoding results.

The published reports on the HRM method claims that it allows for the precise quantification of adulterants by testing the samples in the mixed percentage ranging from 1 to 50% in *Thunbergia laurifolia*, *Phyllanthus* species, Lentils and *Lathyrus cicera* (Ganopoulos et al., 2012; Buddhachat et al., 2015; Singtonat and Osathanunkul, 2015). In the present study, we also tried mixing of different *Senna* species in 1–10% ratio to detect the percentage of contamination of substitute species in commercial samples. HSA samples showed various percentages of mixing of species *S. italica* subsp. *italica*, *S. tora*, *and S. uniflora* in the genuine *S. alexandrina* crude drug through their melting behavior (**Figure 4B**), which was further validated with BLAST and NJ analysis of the sequences from the PCR product of the HRM analysis (**Figure 2**).

Considering the novelty of the HRM technique, the method has successfully emerged as an alternative technological platform for precise identification of herbal plants providing quality control of crude drugs and their finished products (Mader et al., 2011; Sun et al., 2016). Since SCAR marker-enabled discrimination needs species-specific markers for every species and are unable to quantify the extent of contamination (Buddhachat et al., 2015), the HRM technique offers an advantage. Also, the use of DNA barcoding effectively surpassed the previous tools, but is hampered by sequencing costs and time consumption. PCR products of all the *Senna* species for the *ITS* marker amplified 700–800 bp amplicons and could not be differentiated solely on the basis of amplicon size (Supplementary Figure 5). Thus the sequencing of the barcodes followed by processing of melting behavior of amplicons post real-time PCR assays was able to characterize each species through their raw and normalized melting curve analysis. Coupling the barcodes with the HRM method can reduce cost and requires ∼4 h for the process to complete. Moreover, the technique can be analyzed for high-throughput technology in future.

## Conclusion

This study is the first attempt to derive HRM assays based on *ITS1* barcodes toward detection of species composition of *S. alexandrina* raw drug samples currently in the market. The PCR amplification product of all the wild and market samples yielded similar amplicon size with the *ITS* region. Amplifying them in the real-time PCR followed by melting curve analysis produced the characteristic curve for the amplicons at different temperatures. Among the 10 commercial samples sold in herbal market from Bangalore and Tamil Nadu as *S. alexandrina*, six were found to contain *S. italica* subsp. *italica* species and the rest were uncontaminated. The melting curves of these samples were found to be highly complex and unresolved. In view of the increasing demand for natural medicines, the safe supply of quality products is a necessary prerequisite. The ability and performance of the *ITS1* region to discriminate among the *Senna* species by HRM assay resulted in the present proposal of the *ITS1* region as a biomarker in *Senna* adulteration biology. It reflected the accurate phylogenetic relationship among the species and correct botanical identity within the commercial samples, which can be employed to determine species identity, particularly in the absence of characteristic morphological traits. Thus the results presented in this study reveal that DNA barcoding coupled with HRM is highly efficient for authenticating *Senna* herbal products in the market for quality control in the drug supply chain and could be recommended for industrial application.

## Author Contributions

PM and VS contributed to the genotypes sampling and study design. PM conducted the experiments, performed the data and sequence analysis, developed the HRM primers and wrote the main manuscript text. AS and VS critically reviewed the data analysis and manuscript. All authors read and approved the final manuscript.

## Conflict of Interest Statement

The authors declare that the research was conducted in the absence of any commercial or financial relationships that could be construed as a potential conflict of interest.

## Footnotes

1 http://www.boldsystems.org

2 dx.doi.org/10.5883/DS-CIMAP

3 http://www.ebi.ac.uk

4 http://www.phylo.org/

5 http://eu.idtdna.com/analyzer/applications/oligoanalyzer/

6 http://www.endmemo.com/bio/gcdraw.php

## References

[B1] Al-Dakan A. A. Tuffail-Al M. Hannan M. A. (1995). *Cassia Senna* inhibits mutagenic activities of benzo[a]-pyrene, aflatoxin B1, shamma and methyl methanesulfonate. *Basic Clin. Pharmacol. Toxicol.* 77 288–292. 10.1111/j.1600-0773.1995.tb01029.x 8577642

[B2] Balasankar D. Vanilarasu K. Preetha P. S. Rajeswari S. Umadevi M. Bhowmik D. (2013). *Senna* – A medical miracle plant. *J. Med. Plants Stud.* 1 41–47.

[B3] Benson D. A. Cavanaugh M. Clark K. Karsch-Mizrachi I. Lipman D. J. Ostell J. (2013). GenBank. *Nucleic Acids Res.* 41 D36–D42. 10.1093/nar/gks1195 23193287 PMC3531190

[B4] Bertolazzi P. Felici G. Weitschek E. (2009). Learning to classify species with barcodes. *BMC Bioinformatics* 10(Suppl. 14):S7. 10.1186/1471-2105-10-S14-S7 19900303 PMC2775153

[B5] Bown D. (1995). *Encyclopaedia of Herbs and Their Uses.* Moscow: Dorling Kindersley Limited.

[B6] Buddhachat K. Osathanunkul M. Madesis P. Chomdej S. Ongchai S. (2015). Authenticity analyses of *Phyllanthus amarus* using barcoding coupled with HRM analysis to control its quality for medicinal plant product. *Gene* 573 84–90. 10.1016/j.gene.2015.07.046 26188160

[B7] Chen S. Yao H. Han J. Liu C. Song J. Shi L. (2010). Validation of the *ITS2* region as a novel DNA barcode for identifying medicinal plant species. *PLoS One* 5:e8613. 10.1371/journal.pone.0008613 20062805 PMC2799520

[B8] Cirillo C. Capasso R. (2015). Constipation and botanical medicines: an overview. *Phytother. Res.* 29 1488–1493. 10.1002/ptr.5410 26171992

[B9] Cousins M. M. Ou S. S. Wawer M. J. Munshaw S. Swan D. Magaret C. (2012). Comparison of a high-resolution melting (HRM) assay to next generation sequencing for analysis of HIV diversity. *J. Clin. Microbiol.* 50 3054–3059. 10.1128/JCM.01460-12 22785188 PMC3421787

[B10] Cuénoud P. Savolainen V. Chatrou L. W. Powell M. Grayer R. J. Chase M. W. (2002). Molecular phylogenetics of *Caryophyllales* based on nuclear 18S rDNA and plastid *rbcL*, *atpB*, and *matK* DNA sequences. *Am. J. Bot.* 89 132–144. 10.3732/ajb.89.1.132 21669721

[B11] Ewing B. Green P. (1998). Base calling of automated sequencer traces using phred. II. Error probabilities. *Genome Res.* 8 186–194. 10.1101/gr.8.3.186 9521922

[B12] Felsenstein J. (1988). Phylogenies from molecular sequences: inference and reliability. *Annu. Rev. Genet.* 22 521–565. 10.1146/annurev.ge.22.120188.002513 3071258

[B13] Franz G. (1993). The *Senna* drug and its chemistry. *Pharmacology* 1 2–6. 10.1159/000139654 8234429

[B14] Ganopoulos I. Christos B. Madesis P. Kalaitzis P. Tsaftaris A. (2013). Barcode-DNA high resolution melting (BAR-HRM) analysis as a novel close-tubed and accurate tool for olive oil forensic use. *J. Sci. Food Agric.* 93 2281–2286. 10.1002/jsfa.6040 23400707

[B15] Ganopoulos I. Madesis P. Tsaftaris A. (2012). Universal *ITS2* barcoding DNA region coupled with high-resolution melting (HRM) analysis for seed authentication and adulteration testing in leguminous forage and pasture species. *Plant Mol. Biol. Rep.* 30 1322–1328. 10.1007/s11105-012-0453-3

[B16] Gupta R. Pareek S. K. (1995). “*Senna*,” in *Advances in Horticulture*, eds Chadha K. L. Gupta R. (New Delhi: Malhotra Publishing House), 325–336.

[B17] Hall T. A. (1999). BioEdit: a user-friendly biological sequence alignment editor and analysis program for Windows 95/98/NT. *Nucleic Acids Symp. Ser.* 41 95–98.

[B18] Hebert P. D. N. Cywinska A. Ball S. L. deWaard J. R. (2003). Biological identifications through DNA barcodes. *Proc. Biol. Sci.* 270 313–321. 10.1098/rspb.2002.2218 12614582 PMC1691236

[B19] Irwin H. S. Barneby R. C. (1982). The American Cassiinae: a synoptical revision of Leguminosae tribe Cassieae subtribe Cassiinae in the new world. *Mem. N. Y. Bot. Gard.* 35 893-895.

[B20] Jiang C. Cao L. Yuan Y. Chen M. Jin Y. Huang L. (2014). Barcoding melting curve analysis for rapid, sensitive, and discriminating authentication of Saffron (*Crocus sativus* L.) from its adulterants. *Biomed. Res. Int.* 2014:809037. 10.1155/2014/809037 25548775 PMC4274822

[B21] Kalivas A. Ganopoulos I. Xanthopoulou A. Chatzopoulou P. Tsaftaris A. Madesis P. (2014). DNA barcode *ITS2* coupled with high resolution melting (HRM) analysis for taxonomic identification of *Sideritis* species growing in Greece. *Mol. Biol. Rep.* 41 5147–5155. 10.1007/s11033-014-3381-5 24802796

[B22] Kress W. J. Wurdack K. J. Zimmer E. A. Weigt L. A. Janzen D. H. (2005). Use of DNA barcodes to identify flowering plants. *Proc. Natl. Acad. Sci. U.S.A.* 102 8369–8374. 10.1073/pnas.0503123102 15928076 PMC1142120

[B23] Lewis G. P. Schrire B. Mackinder B. Lock M. (2005). *Legumes of the World.* Richmond, VA: Royal Botanic Gardens Kew.

[B24] Li D. Z. Gao L. M. Li H. T. Wang H. Ge X. J. Liu J. Q. (2011). Comparative analysis of a large dataset indicates that internal transcribed spacer (ITS) should be incorporated into the core barcode for seed plants. *Proc. Natl. Acad. Sci. U.S.A.* 108 19641–19646. 10.1073/pnas.1104551108 22100737 PMC3241788

[B25] Mader E. Ruzicka J. Schmiderer C. Novak J. (2011). Quantitative high-resolution melting analysis for detecting adulterations. *Anal. Biochem.* 409 153–155. 10.1016/j.ab.2010.10.009 20946863

[B26] Meistertzheim A. L. Héritier L. Lejart M. (2017). High-resolution melting of 18S rDNA sequences (18S-HRM) for discrimination of bivalve’s species at early juvenile stage: application to a spat survey. *Mar. Biol.* 164:133 10.1007/s00227-017-3162-5

[B27] Mishra P. Kumar A. Nagireddy A. Mani D. N. Shukla A. K. Tiwari R. (2016a). DNA barcoding: an efficient tool to overcome authentication challenges in the herbal market. *Plant Biotechnol. J.* 14 8–21. 10.1111/pbi.12419 26079154 PMC11388846

[B28] Mishra P. Kumar A. Nagireddy A. Shukla A. K. Sundaresan V. (2017). Evaluation of single and multilocus DNA barcodes towards species delineation in complex tree genus *Terminalia*. *PLoS One* 12:e0182836. 10.1371/journal.pone.0182836 28829803 PMC5567895

[B29] Mishra P. Kumar A. Rodrigues V. Shukla A. K. Sundaresan V. (2016b). Feasibility of nuclear ribosomal region *ITS1* over *ITS2* in barcoding taxonomically challenging genera of subtribe Cassiinae (Fabaceae). *PeerJ* 4: e2638. 10.7717/peerj.2638 27994958 PMC5162394

[B30] Morgulis A. Coulouris G. Raytselis Y. Madden T. L. Agarwala R. Schaffer A. A. (2008). Database indexing for production: MegaBLAST searches. *Bioinformatics* 24 1757–1764. 10.1093/bioinformatics/btn322 18567917 PMC2696921

[B31] Newmaster S. G. Grguric M. Shanmughanandhan D. Ramalingam S. Ragupathy S. (2013). DNA barcoding detects contamination and substitution in North American herbal products. *BMC Med.* 11:222. 10.1186/1741-7015-11-222 24120035 PMC3851815

[B32] Osathanunkul M. Madesis P. de Boer H. (2015). Bar-HRM for authentication of plant-based medicines: evaluation of three medicinal products derived from Acanthaceae species. *PLoS One* 10:e0128476. 10.1371/journal.pone.0128476 26011474 PMC4444109

[B33] Palais R. Liew M. Wittwer C. (2005). Quantitative heteroduplex analysis for single nucleotide polymorphism genotyping. *Anal. Biochem.* 346 167–175. 10.1016/j.ab.2005.08.010 16188219

[B34] Posada D. (2008). jModelTest: phylogenetic model averaging. *Mol. Biol. Evol.* 25 1253–1256. 10.1093/molbev/msn083 18397919

[B35] Purushothaman N. Newmaster S. G. Ragupathy S. Stalin S. Suresh D. Arunraj D. R. (2014). A tiered barcode authentication tool to differentiate medicinal *Cassia* species in India. *Genet. Mol. Res.* 13 2959–2968. 10.4238/2014.April.16.4 24782130

[B36] Rama Reddy N. R. Mehta R. H. Soni P. H. Makasana J. Gajbhiye N. A. Ponnuchamy M. (2015). Next generation sequencing and transcriptome analysis predicts biosynthetic pathway of sennosides from *Senna* (*Cassia angustifolia* Vahl.), a non-model plant with potent laxative properties. *PLoS One* 10:e0129422. 10.1371/journal.pone.0129422 26098898 PMC4476680

[B37] Rambaut A. Suchard M. A. Xie D. Drummond A. J. (2014). *Tracer v1.6.* Available at: http://beast.bio.ed.ac.uk/Tracer

[B38] Ratnasingham S. Hebert P. D. N. (2007). BOLD: the barcode of life data system (www.barcodinglife.org). *Mol. Ecol. Notes* 7 355–364. 10.1111/j.1471-8286.2007.01678.x 18784790 PMC1890991

[B39] Reed G. H. Kent J. O. Wittwer C. T. (2007). High-resolution DNA melting analysis for simple and efficient molecular diagnostics. *Pharmacogenomics* 8 597–608. 10.2217/14622416.8.6.597 17559349

[B40] Reed G. H. Wittwer C. T. (2004). Sensitivity and specificity of single-nucleotide polymorphism scanning by high-resolution melting analysis. *Clin. Chem.* 50 1748–1754. 10.1373/clinchem.2003.029751 15308590

[B41] Ronquist F. Teslenko M. van der Mark P. Ayres D. L. Hohna S. Larget B. (2012). MrBayes 3.2: efficient Bayesian phylogenetic inference and model choice across a large model space. *Systemat. Biol.* 61 539–542. 10.1093/sysbio/sys029 22357727 PMC3329765

[B42] Sarwat M. Yamdagni M. M. (2014). DNA barcoding, microarrays and next generation sequencing: recent tools for genetic diversity estimation and authentication of medicinal plants. *Crit. Rev. Biotechnol.* 36 191–203. 10.3109/07388551.2014.947563 25264574

[B43] Schmelzer G. H. Gurib-Fakim A. (2008). *Medicinal Plants*, Vol. 1 Wageningen: Prota Publisher.

[B44] Schmiderer C. Lukas B. Ruzicka J. Novak J. (2015). DNA-Based Identification of *Calendula officinalis* (Asteraceae). *Appl. Plant Sci.* 3:1500069. 10.3732/apps.1500069 26649268 PMC4651632

[B45] Seethapathy G. S. Ganesh D. Santhosh Kumar J. U. Senthilkumar U. Newmaster S. G. Ragupathy S. (2014). Assessing product adulteration in natural health products for laxative yielding plants, *Cassia*, *Senna*, and *Chamaecrista* in Southern India using DNA barcoding. *Int. J. Legal Med.* 129 693–700. 10.1007/s00414-014-1120-z 25425095

[B46] Singh V. (2001). *Monograph on the Indian subtribe Cassiinae.* Jodhpur: Scientific Publishers.

[B47] Singtonat S. Osathanunkul M. (2015). Fast and reliable detection of toxic *Crotalaria spectabilis* Roth. in *Thunbergia laurifolia* Lindl. herbal products using DNA barcoding coupled with HRM analysis. *BMC Complement. Altern. Med.* 15:162. 10.1186/s12906-015-0692-6 26024888 PMC4448308

[B48] Song M. Li J. Xiong C. Liu H. Liang J. (2016). Applying high-resolution melting (HRM) technology to identify five commonly used *Artemisia* species. *Sci. Rep.* 6:34133. 10.1038/srep34133 27698485 PMC5048426

[B49] Sultana S. Ahmad M. Zafar M. Khan M. A. Arshad M. (2012). Authentication of herbal drug *Senna* (*Cassia angustifolia* Vahl.): a village pharmacy for Indo-Pak subcontinent. *Afr. J. Pharm. Pharmacol.* 6 2299–2308. 10.5897/AJPP12.446

[B50] Sun W. Li J. J. Xiong C. Zhao B. Chen S. L. (2016). The potential power of Bar-HRM technology in herbal medicine identification. *Front. Plant Sci.* 7:367. 10.3389/fpls.2016.00367 27066026 PMC4811891

[B51] Sun W. Yan S. Li J. Xiong C. Shi Y. Wu L. (2017). Study of commercially available *Lobelia chinensis* products using Bar-HRM technology. *Front. Plant Sci.* 8:351. 10.3389/fpls.2017.00351 28360920 PMC5352710

[B52] Toi C. S. Dwyer D. E. (2008). Differentiation between vaccine and wild- type varicella-zoster virus genotypes by high-resolution melt analysis of single nucleotide polymorphisms. *J. Clin. Virol.* 43 18–24. 10.1016/j.jcv.2008.03.027 18479962

[B53] Weitschek E. Van Velzen R. Felici G. Bertolazzi P. (2013). BLOG 20: a software system for character-based species classification with DNA barcode sequences-what it does, how to use it. *Mol. Ecol. Res.* 13 1043–1046. 10.1111/1755-0998.12073 23350601

[B54] Wittwer C. T. (2009). High-resolution DNA melting analysis: advancements and limitations. *Hum. Mutat.* 30 857–859. 10.1002/humu.20951 19479960

[B55] Wojdacz T. K. Dobrovic A. (2007). Methylation-sensitive high resolution melting (MS-HRM): a new approach for sensitive and high-throughput assessment of methylation. *Nucleic Acids Res.* 35:e41. 10.1093/nar/gkm013 17289753 PMC1874596

[B56] Wolfe K. H. Li W. H. Sharp P. M. (1987). Rates of nucleotide substitution vary greatly among plant mitochondrial, chloroplast, and nuclear DNAs. *Proc. Natl. Acad. Sci. U.S.A.* 84 9054–9058. 10.1073/pnas.84.24.9054 3480529 PMC299690

[B57] Zhang Z. Schwartz S. Wagner L. Miller W. (2000). A greedy algorithm for aligning DNA sequences. *J. Comp. Biol.* 7 203–214. 10.1089/10665270050081478 10890397

[B58] Zheng S. Jiang X. Wu L. Wang Z. Huang L. (2014). Chemical and genetic discrimination of *Cistanches herba* based on UPLC-QTOF/MS and DNA barcoding. *PLoS One* 9:e98061. 10.1371/journal.pone.0098061 24854031 PMC4031141

