# Supplementary material for: Candidate DNA Barcode Tags Combined With High Resolution Melting (Bar-HRM) Curve Analysis for Authentication of Senna alexandrina Mill. With Validation in Crude Drugs
Source: Front Plant Sci. 2018 Mar 13;9:283. doi: 10.3389/fpls.2018.00283 (PMC5859231; doi:10.3389/fpls.2018.00283)
Supplement: Supplementary file 1 [file Table_1.DOC]

| **Species (Number of individuals)** | **Voucher details** | **BOLD database sample Id** | **GenBank accession numbers** | | | | |
| --- | --- | --- | --- | --- | --- | --- | --- |
|  |  |  | ***rbcL*** | ***matK*** | ***trnH-psbA*** | ***ITS*** | ***ITS2*** |
| *S. alexandrina* (3) | CIMAP-C025 | SAC025 | KY464108 | KY513092 | KY464126 | KY214459 | KY492291 |
|  | CIMAP-C032 | SAC032 | KY464109 | KY513093 | KY464127 | KY427086 | KY492292 |
|  | CIMAP-C039 | SAC039 | KY464110 | KY513094 | KY464128 | KY576674 | KY492293 |
| *S. italica* subsp. *micrantha* (3) | CIMAP-C026 | SAC026 | KY464111 | KY623362 | KY635830 | KY214460 | KY492294 |
|  | CIMAP-C033 | SAC033 | KY464112 | KY623363 | KY635831 | KY576675 | KY492295 |
|  | CIMAP-C040 | SAC040 | KY464113 | KY623364 | KY576681 | KY576676 | KY492296 |
| *S. spectabilis* (3) | CIMAP-C027 | SAC027 | KY464114 | KY513095 | KY576682 | KY214461 | KY492297 |
|  | CIMAP-C034 | SAC034 | KY464115 | KY513096 | KY576683 | KY427087 | KY492298 |
|  | CIMAP-C041 | SAC041 | KY464116 | KY513097 | KY576684 | KY576677 | KY492299 |
| *S. auriculata* (3) | CIMAP-C028 | SAC028 | KY464117 | KY513098 | KY576685 | KY214462 | KY492300 |
|  | CIMAP-C035 | SAC035 | KY464118 | KY513099 | KY576686 | KY427088 | KY492301 |
|  | CIMAP-C042 | SAC042 | KY464119 | KY513100 | KY576687 | KY611897 | KY492302 |
| *S. uniflora* (3) | CIMAP-C029 | SAC029 | KY464120 | KY549326 | KY576688 | KY214463 | KY492303 |
|  | CIMAP-C036 | SAC036 | KY464121 | KY549327 | KY576689 | KY427089 | KY492304 |
|  | CIMAP-C043 | SAC043 | KY464122 | KY549328 | KY576690 | KY611898 | KY492305 |
| *S. tora (3)* | CIMAP-C030 | SAC030 | KY464123 | KY549329 | KY576691 | KY214464 | KY492306 |
|  | CIMAP-C037 | SAC037 | KY464124 | KY549330 | KY576692 | KY427090 | KY492307 |
|  | CIMAP-C044 | SAC044 | KY464125 | KY549331 | KY576693 | KY576678 | KY492308 |
| *S. italica* subsp. *italica* (3) | CIMAP-C031 | SAC031 | KY623359 | KY549332 | KY635832 | KY214465 | KY611894 |
|  | CIMAP-C038 | SAC038 | KY623360 | KY549333 | KY635833 | KY576679 | KY611895 |
|  | CIMAP-C045 | SAC045 | KY623361 | KY549334 | KY635834 | KY576680 | KY611896 |

**Supplementary Table 1 Taxon sampling, BOLD database details and GenBank accession numbers of the reference species generated through this study.**
