# Supplementary material for: Candidate DNA Barcode Tags Combined With High Resolution Melting (Bar-HRM) Curve Analysis for Authentication of Senna alexandrina Mill. With Validation in Crude Drugs
Source: Front Plant Sci. 2018 Mar 13;9:283. doi: 10.3389/fpls.2018.00283 (PMC5859231; doi:10.3389/fpls.2018.00283)
Supplement: Supplementary file 2 [file Table_2.doc]

**Supplementary Table 2 Details of Raw Herb Materials Collected for Analysis.**

| **Particulars** | **Sample Code** | **Voucher Code** |
| --- | --- | --- |
| Novel Nutrients Private Limited, Abbigere, Bangalore | HSA01 | CIMAP-HSA46 |
| Aster Naturals Private Limited, Sahakara Nagar Bangalore | HSA02 | CIMAP-HSA47 |
| Indian Drug Stores, Malleshwaram, Bangalore | HSA03 | CIMAP-HSA48 |
| Primeval Healthcare, Royal Enclave Road, Bangalore | HSA04 | CIMAP-HSA49 |
| Gajalakshmi Exim India Limited, Coimbatore, Tamil Nadu | HSA05 | CIMAP-HSA50 |
| M. Gnanasigamani Nadar Sons, Tuticorin, Tamil Nadu | HSA06 | CIMAP-HSA51 |
| Roshan Trading Company, Tirunelveli, Tamil Nadu | HSA07 | CIMAP-HSA52 |
| Kevina Herbs & Spices Impex, Thoothukudi, Tamil Nadu | HSA08 | CIMAP-HSA53 |
| Rhonda Exports, Madurai, Tamil Nadu | HSA09 | CIMAP-HSA54 |
| Muthu Traders, Tirunelveli, Tamil Nadu | HSA10 | CIMAP-HSA55 |
