# Supplementary material for: Candidate DNA Barcode Tags Combined With High Resolution Melting (Bar-HRM) Curve Analysis for Authentication of Senna alexandrina Mill. With Validation in Crude Drugs
Source: Front Plant Sci. 2018 Mar 13;9:283. doi: 10.3389/fpls.2018.00283 (PMC5859231; doi:10.3389/fpls.2018.00283)
Supplement: Supplementary file 3 [file Data_Sheet_1.PDF]

Supplementary Material

Candidate DNA Barcode-Based High Resolution Melting Curve (Bar-HRM) Analysis for Authentication of *Senna Alexandrina* Mill. With Validation in Crude Drugs

Priyanka Mishra[1], Ashutosh K. Shukla[2] and V. Sundaresan[1,*]

[1]Plant Biology and Systematics, CSIR-Central Institute of Medicinal and Aromatic Plants, Research Centre, Allalsandra, GKVK Post, Bangalore-560065, Karnataka, India

[2]Biotechnology Division, CSIR-Central Institute of Medicinal and Aromatic Plants, P.O. CIMAP, Lucknow-226015, Uttar Pradesh, India

Correspondence:

V. Sundaresan

vsundaresan@cimap.res.in; resanvs@gmail.com

Supplementary Figures

Supplementary Figure 1. Geographical mapping of the *Senna* specimens of this study with voucher details as mentioned in Supplementary Table 1.

Supplementary Figure 2. Strict consensus tree showing the relationship of *Senna* species resulting from maximum parsimony analysis. Tree length = 490, CI = 83%, RI = 93%, RC = 77%. Bootstrap support values below 60% are not shown. For species codes refer to Supplementary Table 1.

Supplementary Figure 3. The specificity of designed HRM primer set ST030HRMF/R using conventional PCR. M Represent 2-Log DNA ladder and Lanes 2-8 correspond to different *Senna* species as coded in Supplementary Table 1.

Supplementary Figure 4. Crude drug sold in processed forms. From original plant to powder form. (a) *S. italica subspecies micrantha.* (b) *S. alexandrina*

Supplementary Figure 5. The specificity of universal *ITS* primer set using conventional PCR. M represent 2-Log DNA ladder and lanes 2-8 correspond to different *Senna* species as coded in Supplementary Table 1.
