# Supplementary figures and images for: Candidate DNA Barcode Tags Combined With High Resolution Melting (Bar-HRM) Curve Analysis for Authentication of Senna alexandrina Mill. With Validation in Crude Drugs
Source: Front Plant Sci. 2018 Mar 13;9:283. doi: 10.3389/fpls.2018.00283 (PMC5859231; doi:10.3389/fpls.2018.00283)

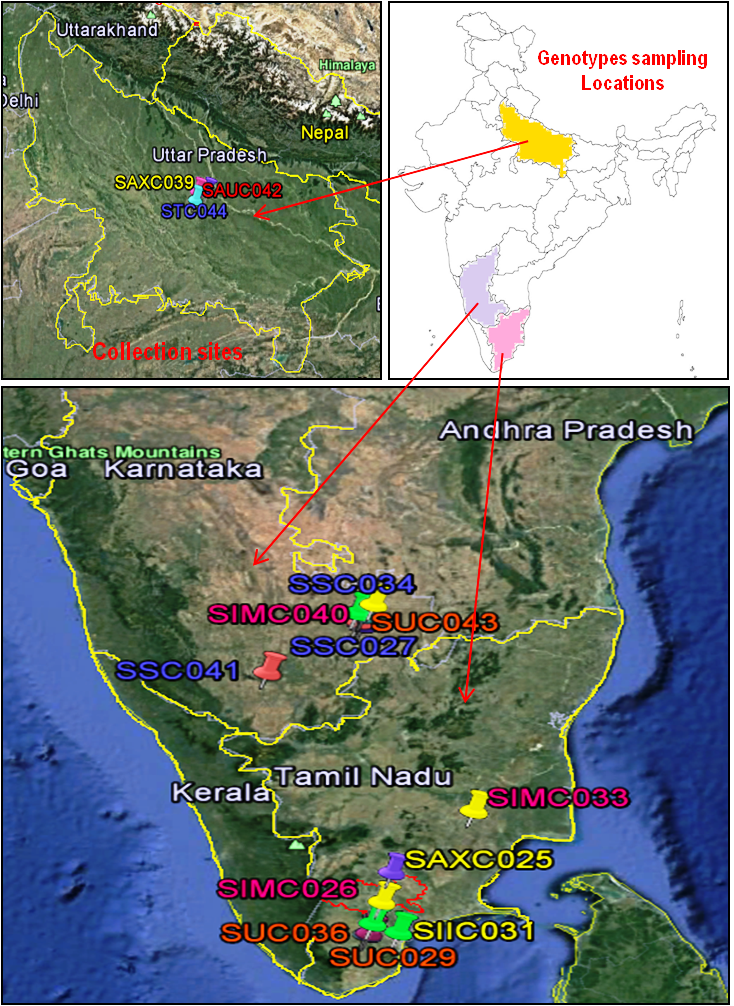

Supplement: Supplementary file 4 [file Image_1.TIF]

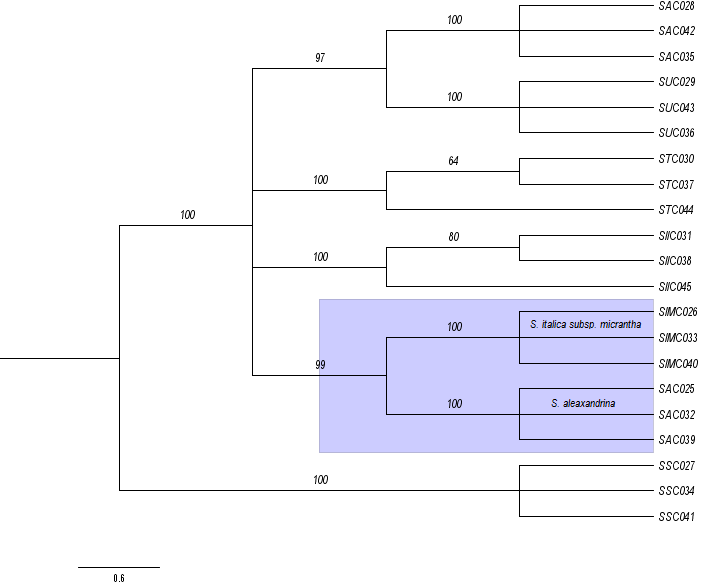

Supplement: Supplementary file 5 [file Image_2.TIF]

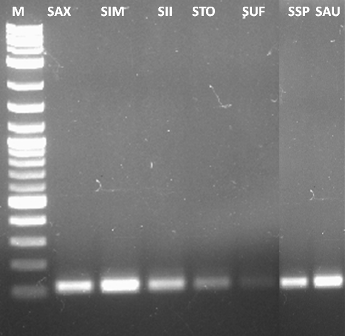

Supplement: Supplementary file 6 [file Image_3.TIF]

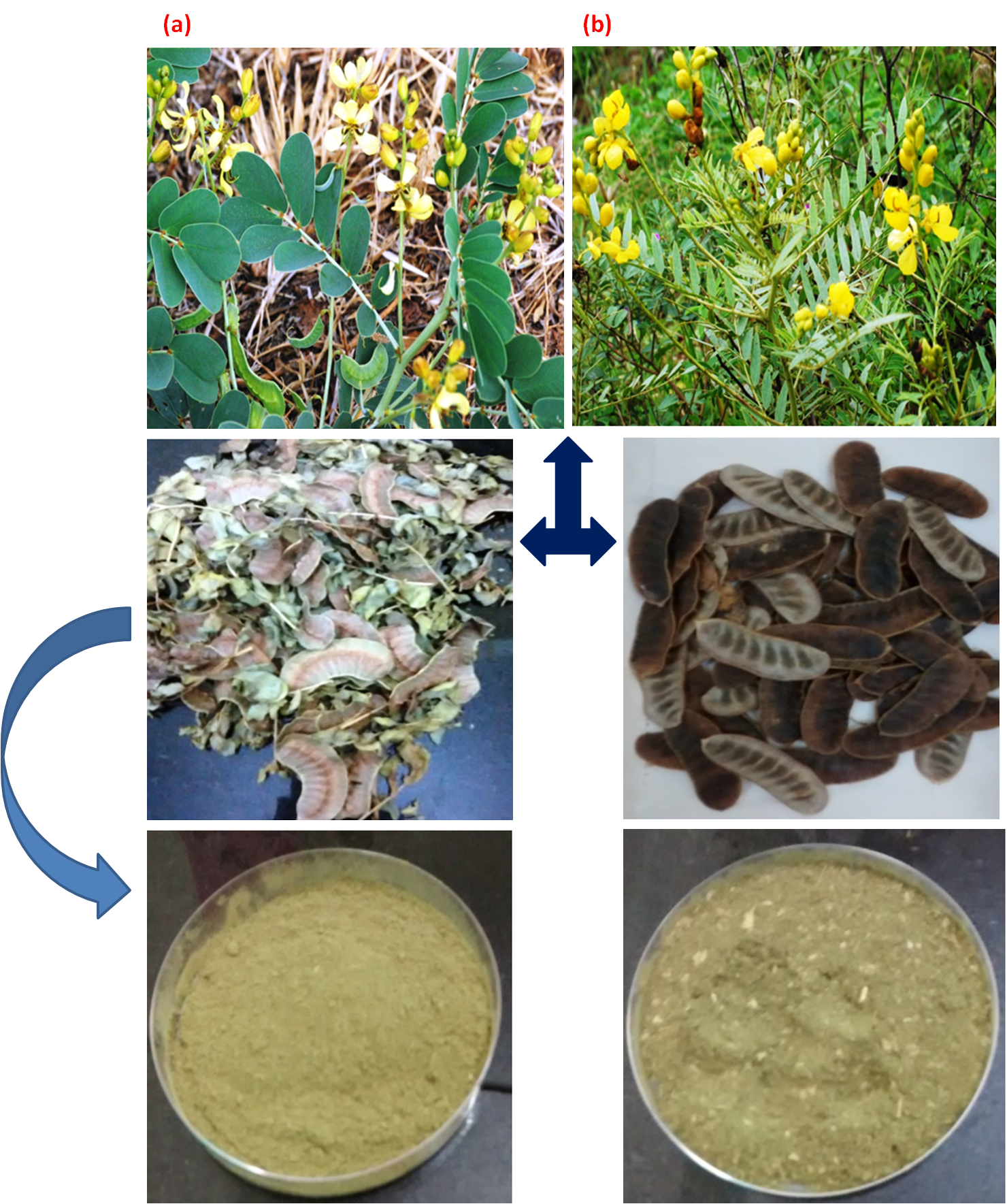

Supplement: Supplementary file 7 [file Image_4.tif]

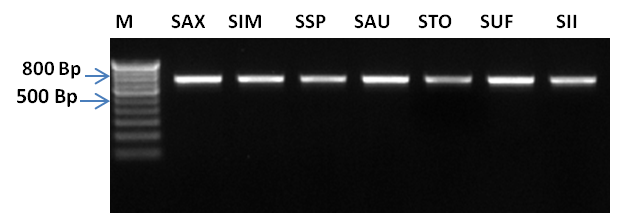

Supplement: Supplementary file 8 [file Image_5.TIF]
